# Dynamic Target Tracking Method Based on Medical Imaging

**DOI:** 10.3389/fphys.2022.894282

**Published:** 2022-05-10

**Authors:** Guofeng Qin, Jiahao Qin, Qiufang Xia, Jianghuang Zou, Pengpeng Lin, Chengkun Ren, Ruihan Wang

**Affiliations:** ^1^ Department of Computer Science and Technology, Tongji University, Shanghai, China; ^2^ Department of Chinese Medicine Rehabilitation, Shanghai First Rehabilitation Hospital, Shanghai, China

**Keywords:** rehabilitation medicine, swallowing angiogra-phy, image analysis, tissue and organ, dynamic target tracking, barium flow

## Abstract

The cross fusion of rehabilitation medicine and computer graphics is becoming a research hotspot. Due to the problems of low definition and unobvious features of the initial video data of medical images, the initial data is filtered and enhanced by adding image preprocessing, including image rotation and contrast enhancement, in order to improve the performance of the tracking algorithm. For the moving barium meal, the discrete point tracking and improved inter frame difference method are proposed; for the position calibration of tissues and organs, the Kernel Correlation Filter (KCF) and Discriminative Scale Space Tracker (DSST) correlation filtering method and the corresponding multi-target tracking method are proposed, and the experimental results show that the tracking effect is better. The two algorithms modify each other to further improve the accuracy of calibration and tracking barium meal flow and soft tissue organ motion, and optimize the whole swallowing process of moving target tracking model.

## 1 Introduction

Traditional video fluoroscopic swallowing study (VFSS) is special X-ray fluoroscopy for the mouth, throat and esophagus while swallowing. Doctors diagnose dysphagia by observing the video sequence, and make a special rehabilitation plan for patients. The doctor’s subjectivity has a great influence on the results in this manual way, so that it is difficult to analyze quantitatively. Also, the doctors need a long term of learning, training and practicing. With the rapid development and iteration of computer hardware and software, the use of computer technology in medical imaging for auxiliary diagnosis is gradually becoming a trend, which is the significance of the research on this subject.

Moving target detection can accurately separate the moving target from the background image, so as to determine the specific location of the target. Moving target tracking is to transfer the detected parameter information to the corresponding module, and the corresponding technology analyzes, processes and counts these parameters. After decades of exploration and research, moving target detection and tracking technology has achieved good results and is widely used in intelligent video surveillance, unmanned driving and other fields.

The data sources of this paper are mainly the video sequences of upper digestive tract. Doctors analyze the swallowing condition of patients by observing the movement of barium meal swallowing in the mouth, throat and esophagus. According to the characteristics of swallowing video sequence, this paper proposes some target tracking algorithms to ensure the accuracy of the algorithm. At the same time, this paper also compares the performance of the traditional algorithm with the algorithm proposed in this paper to ensure the optimization and upgrading of the performance of the algorithm proposed in this paper. It will be introduced in the following chapters.

This paper discusses different tracking targets and proposes the following algorithms:

1) For the motion trajectory of barium meal, discrete point tracking and improved inter frame difference method are proposed in this paper.
2) For the tissues and organs involved in the swallowing process, Kernel Correlation Filter (KCF) combined with barium meal correction is used to deal with the changes of location information.
3) For the change of the number of multiple tissues and organs and barium meal, the multi-target tracking method is applied to update the motion trajectory.

## 2 Material and Methods

### 2.1 Research Status of Target Tracking

In the field of computer vision research, motion target detection and tracking has been the focus of scientific workers. Motion target tracking on VFSS is to determine the position and the shape of the target in each frame. Currently, many studies on target tracking in medical images are mainly based on the results of medical image segmentation. Segmentation methods and comparisons based on thresholds, regions and edge are proposed (Jayaram, 1996; Alin Achim, 2003; Wei Weibo, 2009; Li Mingzhong, 2016). Correspondingly many target-tracking methods have been proposed for medical image processing and analysis.

The adaptive background mixing model proposed by Stauffer (1999) establishes a Gaussian mixture function model for each pixel, and updates the model with linear approximation. Compared with the non adaptive background modeling method, it does not need manual initialization, and has good effects in light changes, recognition of slow-moving objects, the entry and exit of objects in the scene, complex area tracking and repeated movement of scene elements. The ViBe algorithm proposed by Barnich (2009). It is mainly composed of three aspects: working principle, initialization method and update strategy. ViBe’s update strategy is to recognize a pixel from the foreground to the background for the first time. It does not operate on it, but counts the state. When it is detected as a foreground for many times in a row, it is updated as a background point. Vibe’s effect is also very good. It is very stable for the effects of illumination change and jitter. Moreover, due to the small amount of calculation, the efficiency is also very high. Subsequently, Van Droogenbroeck (2012) improved some details of ViBe to improve the performance of the algorithm. For example, reducing the parameters of update factor can speed up the speed of background learning; Fill the foreground of small background blocks to prevent misjudgment; When the gradient of the background module is large, the neighborhood update is suppressed, which is suitable for the case where the moving target block is stationary.

There are three types of motion tracking methods: point-based, contour-based and kernel-based tracking methods, the most representative methods of each are Bayesian tracking method, deformable tracking model (McInerney and Terzopoulos, 1996; Wang, 2013) and harmonic phase (HARP) algorithm (Lim, 2015). These three methods are widely used for motion tracking in medical images nowadays.

Since the target is in continuous motion, the points with consistent motion characteristics in several frames can be classified as motion target feature points with the rest as background. Then the motion target can be tracked based on these feature points, and typical algorithms include the feature optical flow method (Cao, 2001), the frame difference method, etc., and (Wu and Mao, 2013) also proposed a detection tracking algorithm combining the frame difference method and the feature optical flow method, etc. Many motion tracking methods based on or combined with Bayesian framework have been widely used in recent years. Among the Bayesian tracking methods, the particle filter (Zhang, 2017) is one of the most popular motion tracking techniques in medical image analysis and is used to track the motion of lumbar spine, lung, heart and molecular cells (Lim, 2015). Compared to the Kalman filter (Welch, 1995), particle filters are more powerful in dealing with state variables and non-Gaussian problems with multi-peaked distributions.

The ability of deformable models to exploit multiple image attributes and high-level or global information to improve the robustness of shape recovery is one of the most intensively studied model-based approaches to computer-aided medical image analysis (Johnson, 2015). The widespread acceptance of deformable models stems mainly from their ability to segment, match, and track images using constraints derived from image data as well as knowledge about size and position. For example, many snake models now combine region-based image features with traditional edge-based features (McInerney and Terzopoulos, 1996). Strategies worth further investigation include merging shape constraints into deformable models that are derived from low-level image processing operations such as refinement, medial transformations, or mathematical morphology. A classical approach to improve the robustness of model fitting is the use of multi-scale image preprocessing techniques (Terzopoulos, 1993), possibly combined with multi-resolution deformable models (Bajcsy, 1989).

The HARP algorithm is can execute fast even on a regular computer, and the motion tracking results are relatively accurate, and thus has been widely used by the medical image analysis circles as a standard processing technique for MRI labelling.

Traditionally, template-matching-based methods select the appropriate model to successfully detect and calibrate the target. Without a specific modified adaptation, tracking is reliable only for short periods of time and without drastic deformations. However, in most application cases, the target appearance undergoes significant structural changes over long periods of time due to point-of-view changes, deformations, or occlusions, and motion tracking-based methods (Shi and Tomasi, 1994; Sidenbladh et al., 2000) can handle such appearance changes. However, cumulative motion deviations and rapid visual changes cause the model to deviate from the tracking target. Tracking performance can be improved by imposing object-specific subspace constraints (Black and Jepson, 1998) or by maintaining the model statistical representation (Jepson et al., 2003).

Recent advances in discriminative learning and the opening of large medical databases with expert annotations have made learning-based methods (Wang, 2013; McInerney and Terzopoulos, 1996) attractive in order to achieve robust object detection and tracking in medical imaging. Wang (2013) proposed a robust information fusion method that combines learning-based and conventional methods for fast and accurate detecting and tracking of deformable objects with various applications in the field of medical imaging.

In the field of machine learning and pattern recognition, scholars have set to investigate different feature selection and optimization schemes. However, these algorithms require offline training information about the target or the backdrop. Such information is not always available. In addition, recognition features change as the appearance of the object or the backdrop changes. Therefore, differentiated features need to be selected online. Much work has been done in computer vision to select individual features online (Huang, 2009) or to adjust feature weights (C. Ó,2012), which can be applied to motion tracking of medical images to improve adaptability and robustness.

An important issue that has been neglected in motion tracking algorithms is the integration of contextual information in medical images. Contextual information has been extensively studied in image and video understanding. In fact, many psychophysical studies have shown the importance of the environment to the human visual system. With the advancement of machine learning methods, contextual information will play an increasingly important role in future visual tracking studies. Under this circumstance, only (Mountney, 2008) proposed a new technique for accurate tracking of deformed tissue organs based on context-specific feature descriptors that can adapt to the tissue environment and identify the most discriminative tracking information. A tracking method that uses contextual information to merge general constraints on object shape and motion typically performs better than tracking methods that do not utilize this information. This is because a tracker that is designed to provide the best average performance in a variety of scenarios may not be as accurate in one given scenario as a tracker that is designed for the characteristics of the scenario.

### 2.2 Pre-processing

#### 2.2.1 Image Rotation

Image preprocessing is image rotation. The purpose is to establish a new coordinate system based on the spine in each frame, maintain the fixation of the body, and better judge the movement of barium meal relative to various tissues and organs. The rotation center of the image is $O\left(x_{c},y_{c}\right)$ , and there is a pixel point $P_{0}\left(x_{0},y_{0}\right)$ , P(x,y) is the point corresponding to $P_{0}$ rotated around point O clockwise with angle θ . Move the origin of coordinate system to the point of O. Rotate it around O with angle θ . Move it back to the original place.

$$A=\left[\begin{array}{ccc} 1 & 0 & -a\\ 0 & 1 & -b\\ 0 & 0 & 1 \end{array}\right],B=\left[\begin{array}{ccc} \cos\theta & \sin\theta & 0\\ -\sin\theta & \cos\theta & 0\\ 0 & 0 & 1 \end{array}\right],C=\left[\begin{array}{ccc} 1 & 0 & a\\ 0 & 1 & b\\ 0 & 0 & 1 \end{array}\right]$$ (1)

**FIGURE 1:**
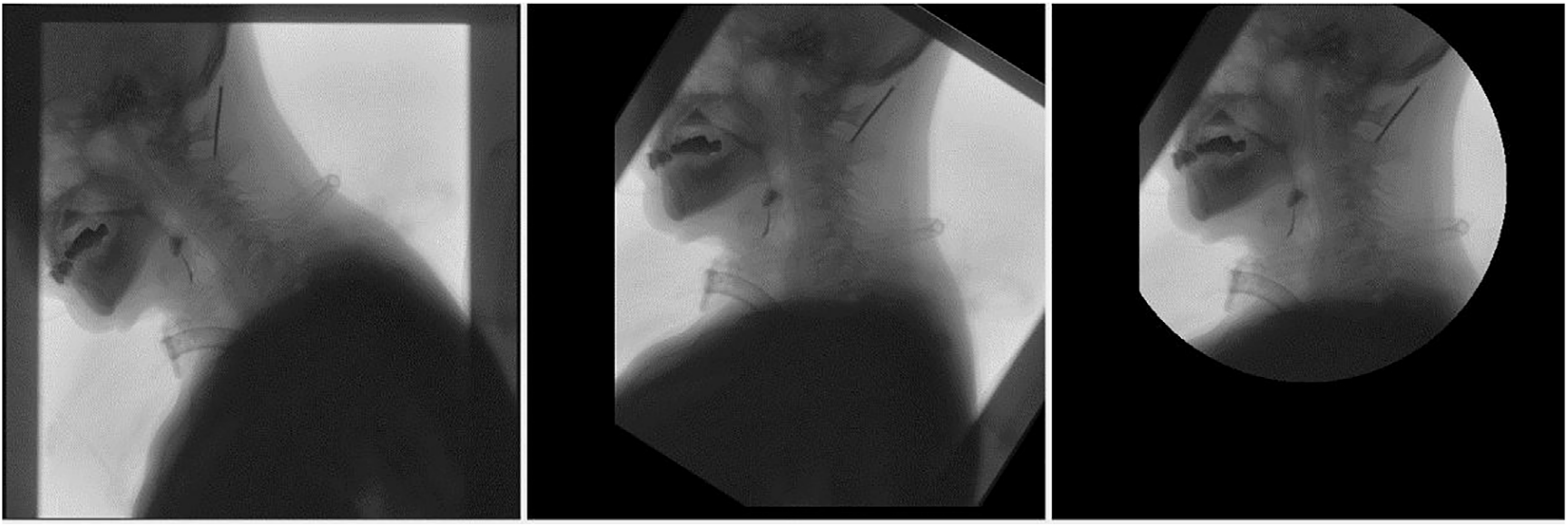
Rotation renderings of pictures.

Thus, $P=CBAP_{0}$ , and the transformation matrix should be C⋅B⋅A . In this paper, after performing the above rotation we added a circular area to specifically label the observed area (Figure 1).

#### 2.2.2 Watershed Segmentation

**FIGURE 2:**
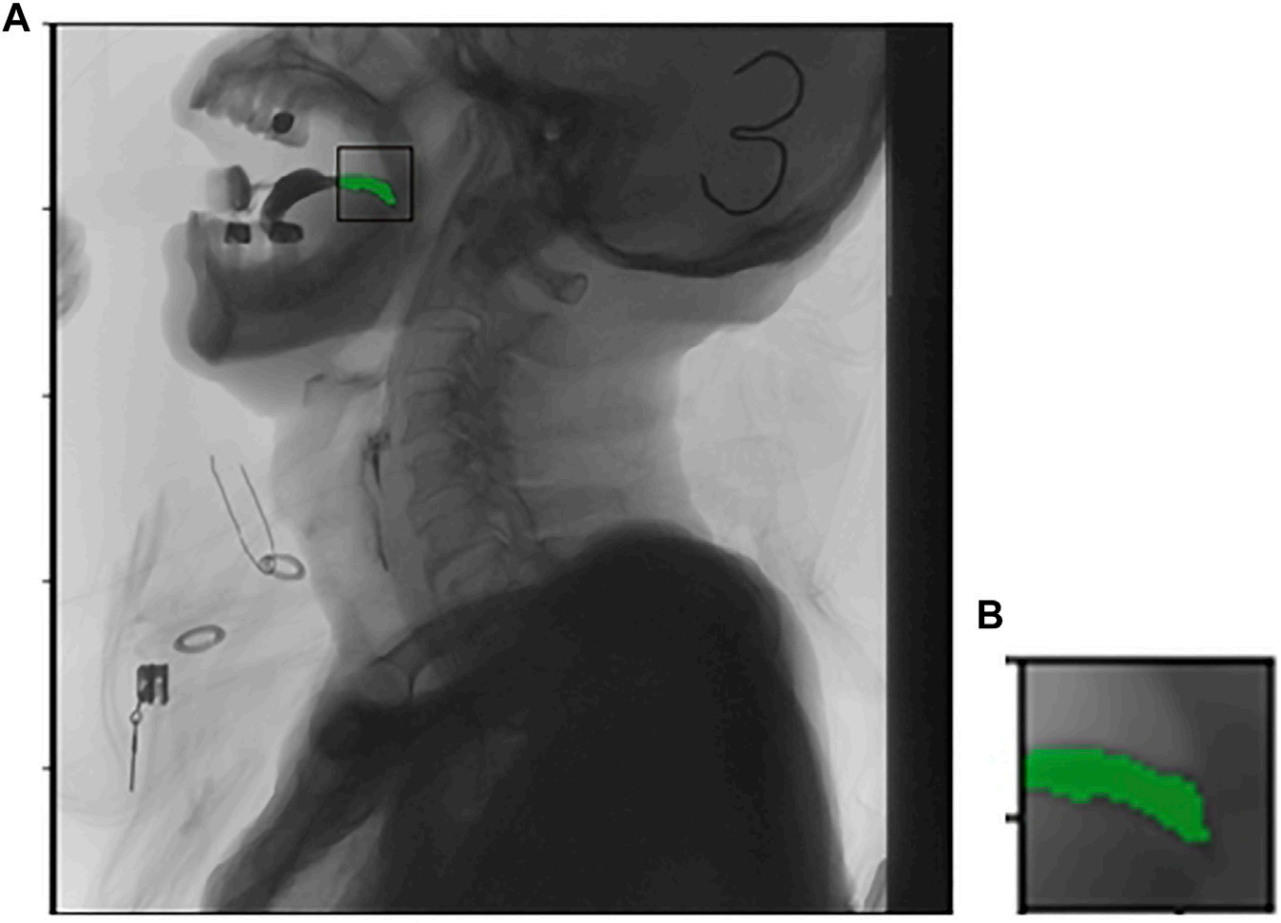
Picture **(A)** is the original imagine, picture **(B)** is the targeted area intercepted.

**FIGURE 3:**
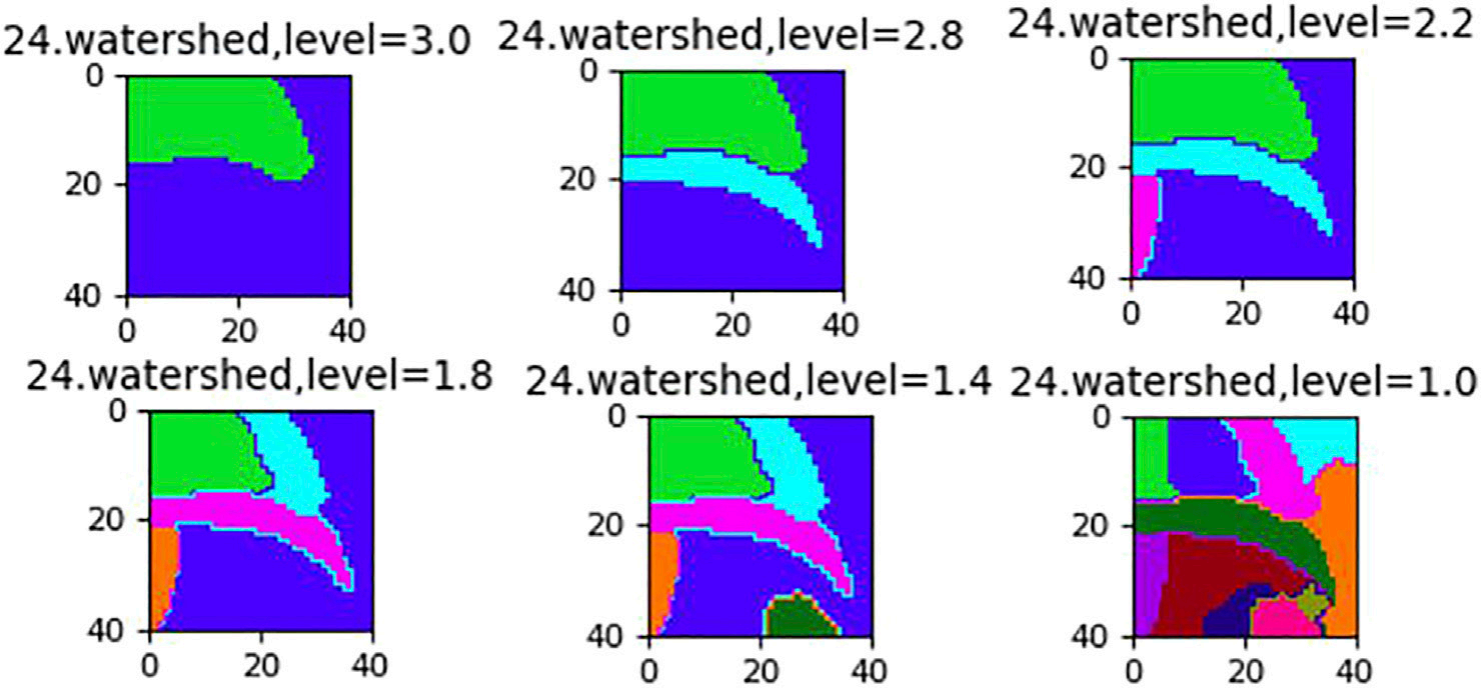
The effect of different segmentation levels.

Watershed segmentation is a powerful and fast method for contour detection and region based on segmentation. We can get a better segmentation effect by dynamically adjusting the segmentation level. In order to eliminate the influence of local minimum caused by noise or quantization error in the last result, the gradient of the original image is calculated as preprocessing, and then watershed transform is applied. Different segmentation levels have different results in Figures 2, 3.

#### 2.2.3 Contrast Stretching

**FIGURE 4:**
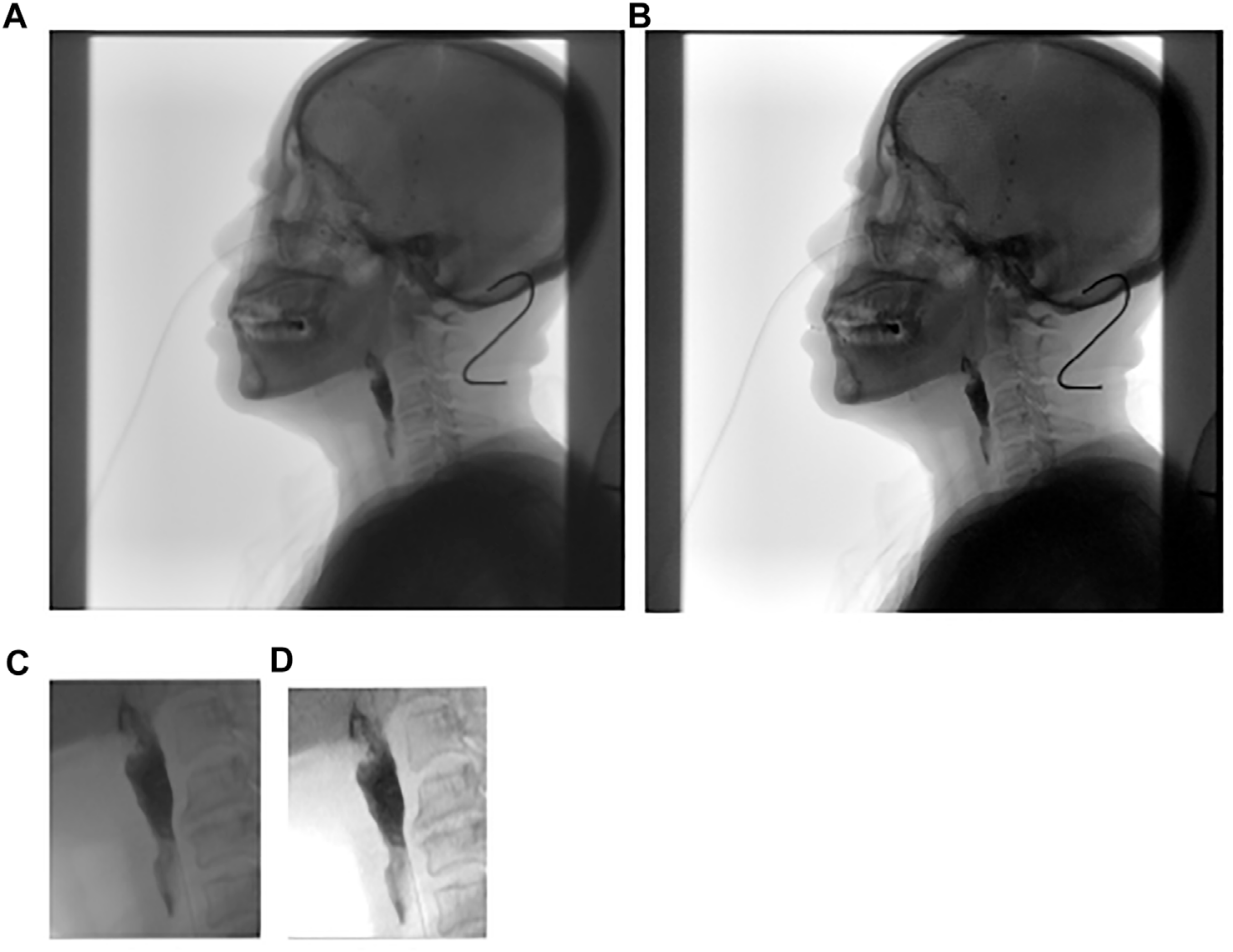
**(A)** is the original image; **(B)** is global contrast stretching; **(C)** is part of **(A)** and **(D)** is local contrast stretching.

In the medical images of this paper, the global contrast enhancement can not obtain satisfactory results. The local difference of pixels is small, and the pixels with large gray difference are often irregularly scattered in all corners, and the images with similar gray levels tend to gather together. So the global contrast stretching is needed. Set the lower limit and upper limit of pixel value $O_{\min}$ and $O_{\max}$ for the image. Find the lowest and highest pixel values $N_{\min}$ and $N_{\max}$ . And scale each pixel P with the following function. The value less than 0 is set to 0, and the value more than 255 is set to 255 in Figure 4​

$$P_{out}=\left(P_{in}-N_{\min}\right)\left(\frac{O_{\max}-O_{\min}}{N_{\max}-N_{\min}}\right)+O_{\min}$$ (2)

### 2.3 Target Tracking

#### 2.3.1 Discrete Point Tracking

The target tracking scenario in this paper is the trajectory of barium meal in the mouth and throat and the movement of tissues and organs. Because the barium meal has the characteristic of flow, there is no fixed appearance characteristics, and the scale and color depth will always change. It is difficult to detect and track the barium meal using stable color or boundary characteristics. We propose a method of discrete point tracking, the main idea of which is to randomly obtain a number of points in the target area as discrete points, and to expand the area where the discrete points are located in the next frame. After that a partition of the expanded area into watersheds is applied; then find out the gray average of each watershed, and use the proportion of discrete points in the current area to all discrete points and the area of the current watershed as evaluation. Then determine whether the area is the target area or part of the target area. After finding the target area, record the target moving distance and moving direction of the current frame and the previous frame in order that it can be adjusted accordingly in the next area expansion. The following is the specific algorithm.

**FIGURE 5:**
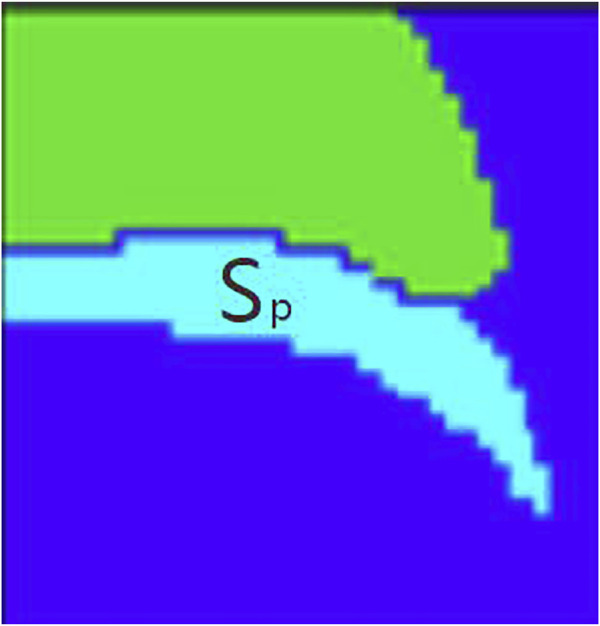
Barium meal area $S_{p}$

The specific steps are to check the target location with a box and find the specific location and contour of the barium meal in the first frame using watershed segmentation. The barium meal area is $S_{p}$ (Figure 5).

Find some random discrete points $P_{r}$ in the barium meal area.

$$P_{r}=random\left(\gamma_{p},S_{p}\right)$$ (3)

In Eq. 3, $\gamma_{p}$ is the ratio of the number of discrete points to the number of $S_{p}$ pixel points, the default of which is set 0.1 in this paper. Then calculate the average grayscale value $g_{avg}$ .

$$g_{av\text{g}}=\frac{1}{n}\sum_{i-1}^{n}S_{p_{i}}$$ (4)

In the next frame, expand the rectangular box by 1.2 times. Then divide the area inside the rectangular box into several regions using the watershed segmentation method in the next frame, with the initial segmentation level c set to 4, and then get all the segmented regions $seg_{1},\cdots seg_{n}$ .

$$U_{i=1}^{n}{seg}_{i}=f_{s}\left(c,img,box\right)$$ (5)

In Eq. 5, “ img ” and “ box ” stand for the original image and the rectangular box, $f_{s}$ is the watershed segmentation function. For any $seg_{i}$ in any set $seg_{1},\cdots seg_{n}$ , it will be judged whether it is a area that meets the requirements. If so, it will be saved. If not, it will be judged whether the proportion of its area size to the rectangular box exceeds a certain threshold value $\beta_{img}$ . If so, the segmentation level will be lowered and the above formula will be recalculated to get a new $seg_{1},\cdots seg_{n}$ ; If not, the current $seg_{i}$ will be processed to determine whether to continue to lower the segmentation level c and subdivide it into more regions. The judging is based on proportion of the number of discrete points in the total number, the ratio of the number of discrete points in $seg_{i}$ and the difference between the average gray level in the region. Then calculate the weighted sum of the three.

$$Eva=\theta_{0}+\theta_{1}\frac{\sum f_{p}\left({seg}_{\text{i}}\right)}{\sum P_{r}}+\theta_{2}\frac{\sum f_{p}\left({seg}_{i}\right)}{\sum se\text{g}}+\theta_{3}\left|gray\left(seg\right)-{\text{g}}_{\text{a}\text{v}\text{g}}\right|$$ (6)

In Eq. 6, $f_{p}$ is a function to solve the number of discrete points in $seg_{i}$ . When the value of Eva not included in $\left[E_{0},E_{1}\right]$ , it means that the judging criterion is not satisfied, the segmentation level c is lowered for $seg_{i}$ , and the watershed segmentation is continued to be refined and judged. With the continuous subdivision of seg , when the segmentation level is low enough, it is no longer possible to obtain effective information by continuing the subdivision, and this is the time the threshold segmentation method is applied to the area.

$$seg\to \{\begin{array}{c} G\left(seg\right)\\ Eva\left(se\text{g}\right)\cdots while\left(c>c_{\min}\right) \end{array}$$ (7)

In Eq. 7, G(seg) is the threshold segmentation process, specific inference value is:

$$G\left(se\text{g}\right)=\begin{array}{c} 1\cdots while(gray\left(seg_{i}\right)in\left[g_{0},g_{1}\right]\\ 0 \end{array}$$ (8)

Connect the eligible together for open-close operation, get the eligible target area, calculate the center point of the area, and perform coordinate operation with the center point in the previous frame, we can get the direction of motion and distance of the present for the previous frame, the value can be used as the initial position of the rectangle frame position in the next frame.

$${position}_{predict}=position+\vec{P_{a}P_{b}}$$ (9)

#### 2.3.2 Inter-frame Differential Method

The discrete point tracking is easy to lose the target once a violent position movement occurs. In order to solve this problem, based on the classical inter-frame difference method, an improved inter-frame difference method is proposed.

1) Classical inter-frame differential method

When the position of the target changes in the video sequence, there will be an obvious difference between two adjacent frames. We subtract the pixels in the two frames to get the difference, and then take the absolute value. Now we can judge whether it is less than the set threshold, so as to further analyze the motion of the target.

$$R\left(x,y\right)=\{\begin{array}{c} 1,if\left|n\left(t\right)-n\left(t-1\right)\right|>m\\ 0,others \end{array}$$ (10)

In Eq. 10, R(x,y) is the difference image between two frames separated by a certain number of frames, n(t) and n(t−1) are the images at moments t and t−1 . m is the threshold value selected for binarization. R(x,y)=1 means the current pixel belongs to the foreground, and R(x,y)=0 means the current pixel belongs to the background.

The advantage of the algorithm is that it is easier to implement, faster with low latency, and does not be affected by changes in light. The disadvantage is that the results are not accurate enough, more sensitive to the threshold value of segmentation, and the target is prone to internal voids.

2) Improved inter-frame differential method

**FIGURE 6:**
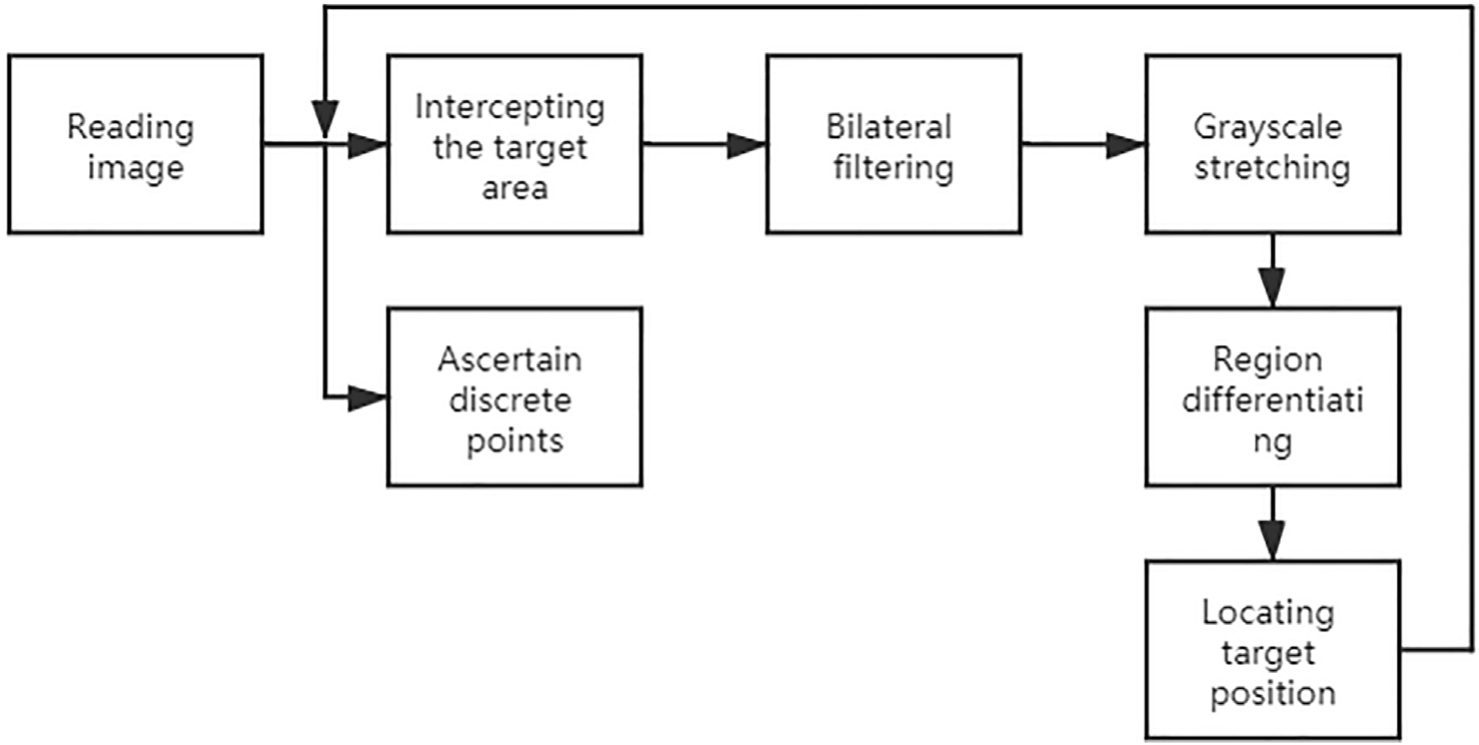
Flow chart of the improved inter-frame difference method.

In this paper, we propose an improved inter-frame differential method (Figure 6), which uses a combination of watershed segmentation, discrete point tracking, and dynamic tracking frame (rectangular region containing the target region) to improve the shortcomings of the differential method.

$$d\_frame\left[x,y\right]=\{\begin{array}{c} 0,d\_frame\left[x,y\right]<T\_diff\\ 1,d\_frame\left[x,y\right]\geq T\_diff \end{array}$$ (11)

At this time, the points in seg are projected to the position of the original image of the current frame. If the point in Image [x,y] corresponding to the original image is higher than the threshold T_p , set seg[x,y]=1 , indicating that this point is a background point to be subtracted in the following steps, otherwise set seg[x,y]=0 , naming the matrix d_seg ; Add up the region of interest seg_p and d_frame and then subtract d_seg to get the region of interest seg_c in the current frame.

seg_c=(d_frame+seg_p)−d_seg (12)

**FIGURE 7:**
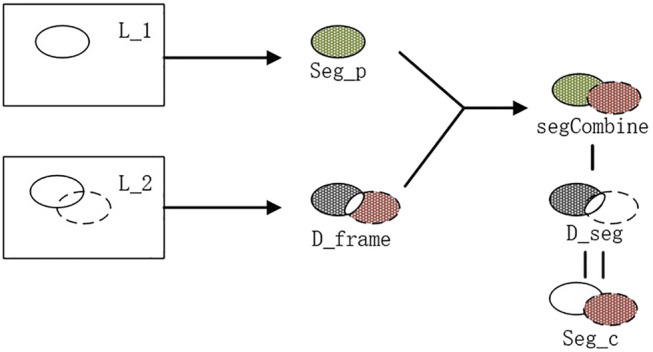
Steps of the inter-frame differential method.

Using this method (Figure 7) for inter-frame differencing, the effect of residual images can be well eliminated, and combined with watershed segmentation, the contours and regions of the moving target can be obtained more accurately.

**FIGURE 8:**
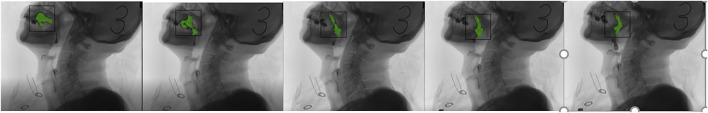
The result of the inter-frame differential method.

Compared with processing the whole image, bilateral filtering of the target region can effectively reduce the impact of noise on image processing. Gray scale stretching makes the span of regions with similar gray scale increase, which is easy to determine and segment. Then the pre-processed image is subjected to improved inter-frame differencing, and the target region obtained is subjected to the open and close operation in morphology to remove the voids and line regions, and the location of the target is determined by combining the discrete point location and grayscale information (Figure 8).

#### 2.3.3 EGMM

The full name of EGMM is Enhanced Gaussian Mixture Model. Gaussian Mixture Model (GMM) refers to the linear combination of multiple Gaussian distribution functions. Theoretically, GMM can fit any type of distribution. It is usually used to solve the situation that the data in the same set contains multiple different distributions, or the same type of distribution with different parameters, or different types of distribution. EGMM is a non-parametric method.

The kernel estimation is using one fixed kernel size D for the whole density function which might not be the best choice. It is called “balloon estimator”. It adapts the kernel size at each estimation point $\vec{\text{x}}$ . We can increase the kernel width D for each new point $\vec{\text{x}}$ until a fixed amount of data k is covered, rather than trying to find the global optimal D. In this way we get large kernels in areas with a small number of samples and smaller kernels in the densely populated areas.

#### 2.3.4 ViBe

The ViBe is a background modeling method proposed by Olivier barnich, 2011 (Zivkovic, 2006; Barnich, 2009). In the traditional background subtraction, building the background model requires large number of video frames, but it can build the background model with only one frame of adjacent pixels. It uses the first frame to initialize the background model and randomly update the corresponding background pixels in time and space, which not only speeds up the calculation speed, but also maintains high accuracy, but also improves the anti-interference ability. Vibe algorithm can be divided into three steps: background initialization, foreground detection and background update.

1) Background initialization

ViBe algorithm randomly selects N pixels in each pixel and its field for modeling according to the similar pixel values of each pixel and its adjacent pixels, and obtains the initialized background model after the same processing for each pixel. The background model has the following meanings:

$$M=\left\{v_{1},v_{2}...v_{n}\right\}$$ (13)

In Eq. 13, n represents the size of sample space; $v_{i}\left(i=1,2,\cdots n\right)$ represents the pixel value corresponding to n pixels.

2) Foreground detection

**FIGURE 9:**
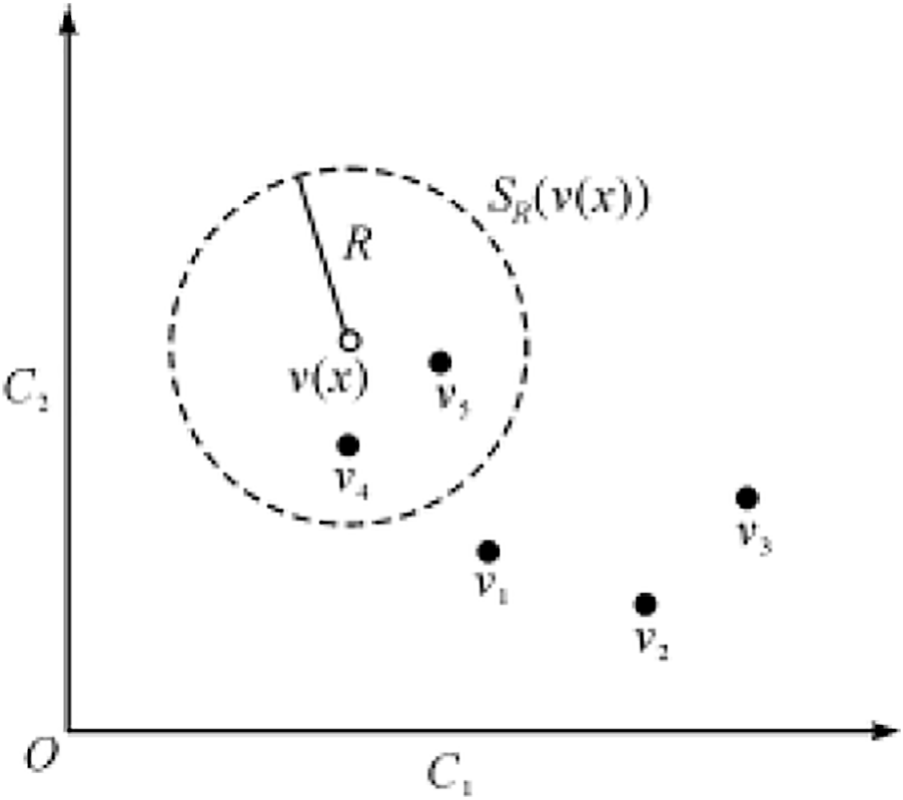
Comparison between current frame and background.

After initializing the background, each new pixel v(x) in a frame is in the Euclidean space $S_{R}\left(v\left(x\right)\right)$ with itself as the center and R as the radius, as shown in Figure 9, where $C_{1}$ and $C_{2}$ are the components of two-dimensional color space $\left(C_{1},C_{2}\right)$ . Calculate the number of pixel intersections n(x) between the space and its background model M(x) , and define the threshold $n_{min}$ of the minimum number of pixel intersections. When n(x) is greater than or equal to the threshold $n_{min}$ , it is determined that the new pixel is the background pixel (recorded as g(x)=0 ); Otherwise, it is determined that the new pixel is a foreground pixel (recorded as g(x)=1 ). The foreground segmentation formula is as Eq. 14.

$$g\left(x\right)=\{\begin{array}{c} 0,n\left(x\right)\geq n_{\min}\\ 1,n\left(x\right)<n_{\min} \end{array}$$ (14)

3) Background update

In the process of updating the vibe background model, due to the strong statistical correlation between pixels and adjacent pixels, when a pixel is detected as a background pixel, the adjacent pixels are likely to be considered as background pixels. In addition, the relationship between the current pixels and their historical samples should also be considered in the process of vibe background update. Therefore, the vibe background update strategy includes time and space update. This is a random protection update strategy, which can integrate the foreground objects that stop suddenly or stay for a long time into the background model.

#### 2.3.5 Correlation Filtering

Correlation filtering applied in signal processing at first, used to describe the relationship between the two signals, or similarity. The basic idea of correlation filtering tracking is: given a filter template, and use the template with the candidate target area for relevancy computation. Then the greatest location of the output response is the goal of the current frame.

y=x⊗m (15)

In Eq. 15, y represents the output of the response, X represents the input image, and m represents the filtering template. It can be converted into a less computable dot product by using related mathematical theorems.

$$\hat{y}=\hat{x}\cdot {\hat{m}}^{*}$$ (16)

In Eq. 16, $\hat{y}$ , $\hat{x}$ , $\hat{m}$ are respectively the Fourier transform of y, x, m. The task of correlation filtering is to find the optimal filtering template.

How to find the best template and the fastest computing method for the scenario in this paper? Firstly, this paper will briefly introduce the research work of some scholars in the field of correlation filtering. Then, in the next chapter, the algorithm model suitable for the scene in this paper is proposed and compared with the classical correlation filtering algorithm.

1) MOSSE

The Minimum Output Sum of Squared Error filter (MOSSE) is the first work of correlation filter tracking (Bolme, 2010). In this paper, the author mentioned that in order to reduce the computation and speed up the response, the convolution operation was changed into dot multiplication operation by FFT.

2) CSK

CSK extends dense sampling on the basis of MOSSE (Henriques, 2012). Dense sampling increases the number of samples without adding too much memory by shifting the image vector like a cyclic matrix. CSK also adds the kernel technique, which can complete the calculation of high-dimensional space in low-dimensional space, and avoid dimensional disaster, improve the speed of sample classification in high-dimensional feature space.

3) KCF

The full name of KCF is Kernel Correlation Filter. It was proposed in Luo, 2014 and caused quite a stir at that time. KCF is a perfection of CSK.

KCF expands HOG features on the basis of CSK, which can replace the original feature to achieve better effect. HOG features which is used widely, in simple terms is to block of the input image, into a cell. After blocking into the smallest unit cell, calculate level gradient and vertical gradient. i×i cells are normalized into a block. In this way we can ignore some large pieces of the non-edge information inside the plane. Also the influence of illumination can be reduced. The advantage of HOG feature is that it uses the vector between pixels as the feature, which means that the brightness and shade of global illumination have limited influence on it, and the robustness is strong. HOG feature is more sensitive to local texture.

4) DSST

Discriminative Scale Space Tracker (DSST) is the improvement on the basis of MOSSE, mainly in response to scale changes, through the two filters tracking the location and scale changes respectively. The first is location filter. It detects target center panning, and determines the new target position; The other is the scale filter, which uses MOSSE of HOG feature to train another scale correlation filter. It is responsible for detecting the target scale change and performing scale estimation. The scale filter only needs to detect the best matching scale without caring about the translation.

DSST replaces the original gray feature with HOG feature, which can describe the target feature better (Danelljan, 2014). The author subsequently made improvements on the basis of DSST. In 2017, an accelerated version of DSST (Danelljan, 2017) was proposed. The main work was to further optimize the algorithm, and the speed was significantly improved. There are two main ideas for improvement: one is to use the correlation difference to improve the accuracy of force; the other is to reduce the feature dimension through principal component analysis.

Based on the above classical algorithm, this paper cleverly combines KCF and DSST to locate the target position more accurately, and uses watershed segmentation to correct the size and edge information of the target.

When the patient is swallowing, there may be a doctor’s assistance, which will cover the patient to a certain extent. The patient swings back and forth when swallowing, and the head may move out of the acquisition area, reflecting incomplete swallowing in the image, which makes it more difficult to keep accurately and continuously track on the patient’s swallowing. The data presented in this paper are mostly for the patient’s side swallowing process. The size of the tissues and organs shows little change. We can use KCF nuclear-related filter to process the tracking process of tissues and organs. The shade and disappear problems can be solved by DSST. The event of a block and disappear can be understood as the scale of the target has changed. Then a scale filter is joined, used to detect the block and disappear event. So we designed two filters, a position filter and a scale filter.

5) Position Filter

When designing the position filter, the first frame and its trace box is used to initialize the position tracker. The sub-window is obtained from the image by the trace box, which is filled with assignment and detected by the feature. The target of the current frame is detected, and the Gaussian correlation function is obtained first.

$$k=f_{gaussiancorrelation}\left(x,z\right)$$ (17)

In Eq. 17, z is the training of the previous frame, and x is the feature of the current frame at the current scale. The response graph is obtained by the discrete Fourier transform.

$$res=f_{real}\left(fftd\left(f_{cm}\left(\hat{\alpha },fftd\left(k\right)\right)\right)\right)$$ (18)

In Eq. 18, $\hat{\alpha }$ is the weighted average of the filtering template related to frequency domain. There are two channels, real part and imaginary part. fftd is fast discrete Fourier transform. $f_{cm}$ is the product of two complex numbers. $f_{real}$ is the real part of the complex. The difference of amplitude is used to locate the position P and the peak $P_{value}$ , which can easily calculate the deviating from the displacement of the center of the sampling. Then based on the current frame, the target position is updated. Before this, whether the target dimension change need to be detected, and the scale filter need to be introduced.

6) Scale Filter

The scale size of the target position in the current frame is M×N . Centered around the middle of the P , capture of different scale images. A series of blocks of the scale of the different scales can be got. When searching for n scales, there will be n scale blocks. Then find out its character description for each size block operator. Before detecting the current image scale, we need to obtain scale samples first.

$${fmap}_{i}{=hann}_{i}pca\left(nor\left({map}_{i}\text{,0}\text{.}2\right)\right)$$ (19)

$${map}_{res}=fftd\left({map}_{1}{Lmap}_{n}\right)$$ (20)

In Eqs 19, 20, $map_{i}$ is the region corresponding to different scale factors. nor , pca is the normalized and principal component analysis to reduce dimensions respectively. $hann_{i}$ is the Hamming factor, which represents the intensity of the image block. Then, similar with the position filter, according to the response graph, the maximum response value and image block index are obtained, and the target box scale is updated. Due to the consideration of appearance changes and other situations, we can not only consider the correlation filter from the previous image, but also consider the previous multiple images at the same time, the sum of which is minimum. After the scale update is completed, we will continue to train the sample parameters using the current detection box.

$$\hat{\alpha }=\left(1-\theta \right)*\hat{\alpha }+\theta *\alpha$$ (21)

In Eq. 21, θ is the adaptive linear interpolation factor, α is the filter template of current frame.

The working framework of this paper can be summarized as follows:

Step 1: In the first frame of the imaging video sequence, the initial position of the target is calibrated, the rectangular tracking region is given. And then the filter is initialized;
Step 2: For each subsequent frame, an image block is extracted from the target position of the previous frame for detection; Considering that the scale changes, the image blocks are multiple;
Step 3: Extraction of gray and HOG features;
Step 4: Replace convolution operation with fast Fourier transform class;
Step 5: After Fourier transform, the response spectrum is obtained, and the position with the maximum response value is the position of the predicted target;
Step 6: The position and scale of the target are extracted to train and update the correlation filter.

**FIGURE 10:**
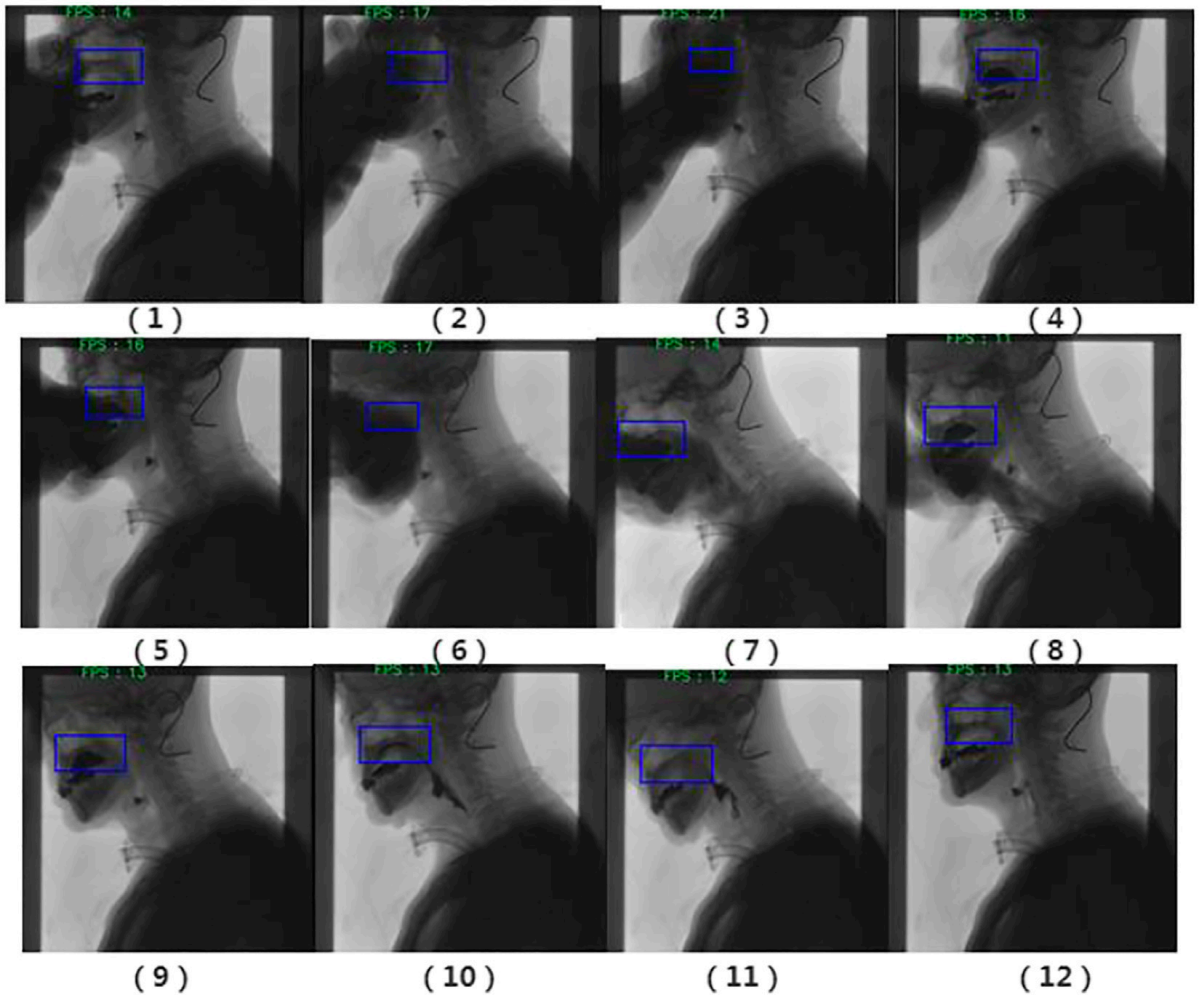
Filtering results. (1) The swallowing stage of the oral cavity; (2) the tracking target is the palate; and (3) Have obvious occlusion; (4) The target is not lost; (5) and (6) Occlusion occurs again; Occlusion and forward leaning; (8) and (9) Barium meal interference; (10) and (11) Barium meal is swallowed into the throat; (12) End of swallowing.

The tracking effect of the algorithm in this paper is as follows:

It can be clearly observed from Figure 10 that occlusion occurs, but the tracker does not lose the target position. With the body leaning forward, the tracking frame can also track the target very well.

### 2.4 Multi-Target Tracking

#### 2.4.1 Analysis of Tracking Model on VFSS

At first, most existing MOT studies can be divided into two categories according to how the target is initialized. The first category is Detection-Based Tracking (DBT). Given a medical imaging video sequence, target detection or motion detection of specific tissues and organs (based on background modeling) is carried out in each frame to obtain target hypothesis, and then batch tracking is carried out to link the detection hypothesis to the video sequence.

The second category is Detection-Free Tracking (DFT), which manually initializes a certain number of tissue and organ targets in the first frame, and then locates these tissues and organs in subsequent frames. DBT features automatic detection of new targets and automatic termination of targets that have disappeared. However, the DFT does not support the new target calibration tracking, meanwhile it does not need training before tracking. The type of targets in this paper is unified, which are all body’s tissues and organs. There is not new target appearing in the video scene. Of course, we can choose DBT. But there are many occlusion cases in this paper, and there are many tissues and organs to be considered and different organ combination also needs a more flexible track scheme. This paper uses the initialization of the DFT. For a video sequence, the doctors can choose the organs free for tracking analysis, or start at different time points to carry out target tracking.

The next is the processing mode. MOT can also be divided into online tracking and offline tracking. The difference between them is whether the subsequent video frames need to be used when the current frame is processed. Online tracking is responsible for the current frame and the previous video sequence information, and the corresponding offline tracking needs to use the information of the future frame to calculate.

It is necessary to obtain the processes and results in time. Online tracking can meet the requirements of the scene in this paper better.

#### 2.4.2 Multi-Target Tracking Model Design

At present, the algorithm that attracts more attention is Simple Online and Real-time Tracking (SORT), a practical method, which is mainly for simple, effective algorithm for multi-target tracking. Sort, as a rough framework, has two algorithms in its core: Kalman filtering and Hungarian matching algorithm. Kalman filtering is divided into two processes: prediction and update. Hungarian algorithm solves an allocation problem. In this paper, when dealing with the relationship between tissues and organs in medical images, it is not necessary to consider real-time data association, but only the independent movement of the target in the whole process. The relationship between multiple targets is calculated separately after the end of tracking. Therefore, the KCF and DSST tracking method in the previous section can be used in the multi-target tracking in this section.

**FIGURE 11:**
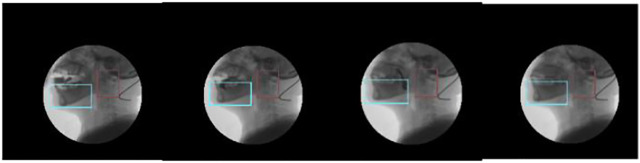
Tracking the chin and the upper part of the spine.

**FIGURE 12:**
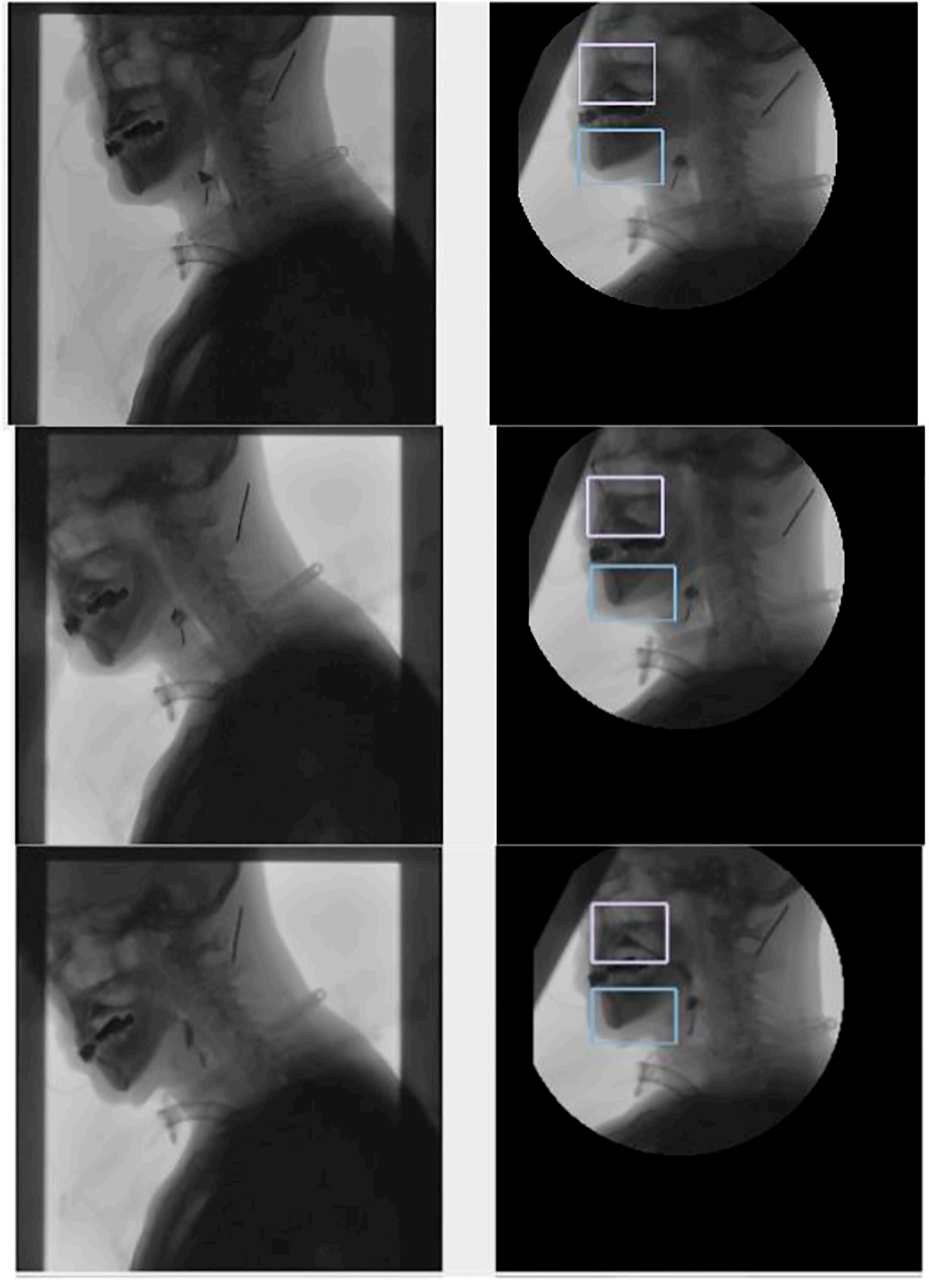
Tracking the chin and nasal cavity. The left one is the original image, and the right one is the tracking results in the coordinate system of the spine.

According to the tracking results (Figures 11, 12), the tracking effect after rotation is more intuitive, and it is more convenient to calculate the position relationship between tissues and organs.

## 3 Result and Analysis

### 3.1 Comparison of Effect of Difference Method

In order to verify the performance of the algorithm better, two algorithms, EGMM and ViBe, are selected in this paper for comparative study. The Enhanced Gaussian Mixture Model (EGMM) (McInerney and Terzopoulos, 1996) is representative and is a deterministic parameter method for background subtraction. The ViBe algorithm (D.-Y., 2009) provides a clearer description of the boundary of the foreground object. In this paper, there is no comparison with classical algorithms (frame difference, optical flow, etc.), because classical algorithms are not sensitive to the change of background, and only a few data have reference value. Data samples were selected from VFSS in the radiology department of the hospital. The details are shown in Table 1.

**TABLE 1:** Sample information.

Type of sample	Quantity	Number of frames per sample
Side View	25	50
Elevation View	5	30

The selection of evaluation index is: average pixel error, which is based on the linear distance between the predicted coordinates and the real coordinates. The smaller the value, the smaller the error. The average overlap rate is expressed by the overlap rate between the target region and the original labeled region. Table 2 shows the performance comparison of the three algorithms in the side view and elevation view.

**TABLE 2:** Performance comparison of algorithms.

Type of data	Algorithm	Average pixel error (APE)	Average overlap ratio (AOR)
Side View	Improved Inter-Frame Difference	9.30	0.78
	EGMM	14.27	0.50
	ViBe	10.11	0.62
Elevation View	Improved Inter-Frame Difference	12.84	0.64
	EGMM	19.51	0.43
	ViBe	25.48	0.33

The speed of the three algorithms is all fast. But ViBe and EGMM are not sensitive to the residual stationary target, and are prone to incomplete recognition, which results that they are not as good as the improved frame difference method in precision and accuracy. For the grayscale images of medical images, the foreground and the background are similar in color, and the latter two algorithms may misjudge the foreground as the background, which results in large errors. Especially in the elevation view, the area of the moving target is small, and there is no obvious movement for a long time. Once the movement occurs, it is violent, which makes the results obtained by the three algorithms not ideal. In this paper, the improved frame difference method considers the residual of moving objects and has great advantages in the result of elevation view. Both EGMM and ViBe need to model the whole background, while the improved frame difference method does not need to save the background information when processing the image in the search box, which greatly saves the memory and improves the performance, and is more suitable for the occasions where real time is required.

### 3.2 Comparison of the Results of Correlation Filtering

Method of this paper has obtained a good effect in the target tracking, followed by comparing with the classic filtering algorithm mentioned above. The reference standards for comparison are accuracy and average speed. This sample set consists of 102 real patient data provided by the hospital. Gaussian filtering and contrast enhancement are performed on these samples. And the same initial frame tracking box is selected; The goal of this study is to provide a diagnostic solution for the hospital. The device simulates the computing power of the hospital device, and is configured as Intel^®^ Core™ i5-7200U CPU @ 2.50GHz × 4+8G without graphics card. The test was conducted in real software. The performance results of each algorithm are shown in Table 3.

**TABLE 3:** The performance comparison among algorithms.

Algorithm	Accuracy (%)	Average Speed(fps)
MOSSE	46.6	138.2
CSK	54.8	78.3
KCF	71.3	24.5
DSST	72.4	19.5
ours	81.3	14.3

The result can be seen that our algorithm has 10 percentage points improvement in accuracy compared with the traditional KCF algorithm, but the average speed decreases due to the more complex algorithm. From the performance of each algorithm, the higher the speed of the algorithm, the lower the accuracy. Considering that the average processing speed of medical scenes is higher than the actual demand.

## 4 Discussion and Conclusion

In this paper, a preliminary study on the target tracking model of medical images has been made. Few scholars at home and abroad have studied the module in the field of imaging video. In the beginning, this paper encountered many problems, including the extraction of patient information from the file, format conversion and subsequent tracking algorithm design. By study of knowledge of medical rehabilitation and computer graphics, we combined them, and began the research of this paper.

For the initial video data, there are problems such as low definition and not obvious features. The tracking algorithm designed in this paper has achieved poor results. By adding the stage of image preprocess, the initial data are screened and enhanced to some extent, including image rotation, contrast enhancement and so on.

For fluid barium meal and less deformed tissues and organs, two methods are proposed respectively. For fluid barium meal, discrete point tracking and improved frame difference method are proposed. For the position calibration of tissues and organs, the correlation filtering method of KCF and DSST, and the corresponding multi-target tracking are proposed, and the tracking effect is proved to be better by experiments. The results of the two can correct each other and optimize the tracking accuracy. The target tracking model is preliminarily established in the process of swallowing. Heap and Hogg, 1996.

## Data Availability

The original contributions presented in the study are included in the article/Supplementary Material, further inquiries can be directed to the corresponding authors.
